# Typhoid Fever?

**Published:** 1869-11

**Authors:** Wm. E. Quine

**Affiliations:** House Physician


### COOK COUNTY HOSPITAL RECORDS.

### SERVICE OF DR. H. A. JOHNSON.

### TYPHOID FEVER?

WM. E. QUINE, M.D., HOUSE PHYSICIAN.

Patrick G. Ireland, laborer, aged 22, admitted Oct. 4, 1869.

*History.*—Delirious on admission, and no previous history obtained. We noticed a dull, besotted expression, suffusion of eyes, hurried breathing, hot, dry skin, soft, quick, frequent pulse, sordes on teeth and lips, dry, brown, fissured tongue, fulness, but not marked resonance, of abdomen, great tenderness of, but no gurgling in, right ileum, and distension of bladder.

*Progress and Treatment.*—Catheterized. Infusism digitalis, to diminish the frequency of the pulse, and to aid warm sponge baths in reducing temperature. Strychnia and acid nitric to stimulate the failing functions of the nervous system.

*October 6th, 1869.*—Bowels moved three times, after the administration of an enema. Urine very scanty and retained in bladder. Potassæ nitras, grs v, every three hours, and catheter introduced twice a day.

*October 8th, 1869.*—Skin not so hot and dry. Urine more copious but is still retained; slight return of consciousness; tongue quite moist and more readily protruded; pulse fuller, stronger, and less frequent. Infusism digitalis discontinued.

*October 10th, 1869.*—Urine continues copious; skin moist, but cold; tongue, also, moist and cold; no movement of bowels since the injectiou was administered on the 6th inst.; pulse a little increased in frequency, and very feeble. Died, October 11th, 1869.

*Autopsy.*—The contents of the abdomen only were examined. Liver, spleen, and mesenteric glands enlarged and softened; hyperæmia of the small and of the ascending portion of the large intestines. Solitary glands and Peyer's patches presented nearly all the stages of destruction and commencing repair; and two points of ulceration extended to the peritoneum.

*Remarks.*—This is the second febrile disease that has come under my observation, in which there was obstinate constipation, entire absence of eruption, and no gurgling in the right iliac fossa, and in which an autopsy revealed very decided "typhoid lesions;" in fact, in the first instance, peritonitis, induced by perforation of the intestine, immediately pıeceded death.

